# Etiology and clinical characteristics of acute viral hepatitis in South Korea during 2020–2021: a prospective multicenter study

**DOI:** 10.1038/s41598-023-40775-5

**Published:** 2023-08-31

**Authors:** Chan Young Jeong, Gwang Hyeon Choi, Eun Sun Jang, Young Seok Kim, Youn-Jae Lee, In Hee Kim, Sung Bum Cho, Jae Hyun Yoon, Kyung-Ah Kim, Dae Hee Choi, Woo Jin Chung, Hyun Chin Cho, Seong Kyun Na, Yun-Tae Kim, Byung Seok Lee, Sook-Hyang Jeong

**Affiliations:** 1 Departments of Internal Medicine, Seoul National University Bundang Hospital, Seoul National University College of Medicine, 82 Gumi-ro 173 Beon-gil, Bundang-gu, Seongnam, 13620 Republic of Korea; 2 Department of Internal Medicine, Soonchunhyang University Bucheon Hospital, Bucheon, Republic of Korea; 3 Department of Internal Medicine, Inje University Busan Paik Hospital, Busan, Korea; 4 Department of Internal Medicine, Jeonbuk National University Hospital, Jeonju, Republic of Korea; 5 Department of Internal Medicine, Chonnam National University Hwasun Hospital, Hwasun, Republic of Korea; 6 Department of Internal Medicine, Chonnam National University Hospital, Gwangju, Republic of Korea; 7 Departments of Internal Medicine, Inje University Ilsan Paik Hospital, Goyang, Republic of Korea; 8 Departments of Internal Medicine, Kangwon National University Hospital, Chunchon, Republic of Korea; 9 Departments of Internal Medicine, Keimyung University Dongsan Hospital, Daegu, Republic of Korea; 10 Departments of Internal Medicine, Gyeongsang National University Hospital, Jinju, Republic of Korea; 11 Departments of Internal Medicine, Jeju National University Hospital, Jeju, Republic of Korea; 12 Seoul Clinical Laboratories, Yongin, Republic of Korea; 13 Departments of Internal Medicine, Chungnam National University Hospital, 282 Munhwa-ro, Jung-gu, Daejeon, 35015 Republic of Korea

**Keywords:** Diseases, Gastroenterology

## Abstract

This prospective, 12-center study investigated the etiology and clinical characteristics of acute viral hepatitis (AVH) during 2020–2021 in South Korea, and the performance of different diagnostic methods for hepatitis E virus (HEV). We enrolled 428 patients with acute hepatitis, of whom 160 (37.4%) were diagnosed with AVH according to predefined serologic criteria. The clinical data and risk factors for AVH were analyzed. For hepatitis E patients, anti-HEV IgM and IgG were tested with two commercial ELISA kits (Abia and Wantai) with HEV-RNA real-time RT-PCR. HAV, HEV, HBV, HCV, Epstein-Barr virus (EBV), cytomegalovirus, and herpes simplex virus accounted for AVH in 78.8% (n = 126), 7.5% (n = 12), 3.1% (n = 5), 1.9% (n = 3), 6.9% (n = 11), 1.2% (n = 2), and 0.6% (n = 1) of 160 patients (median age, 43 years; men, 52.5%; median ALT, 2144 IU/L), respectively. Hospitalization, hemodialysis, and intensive care unit admission were required in 137 (86.7%), 5 (3.2%), and 1 (0.6%) patient, respectively. Two patients developed acute liver failure (1.3%), albeit without mortality or liver transplantation. Ingestion of uncooked clams/oysters and wild boars’ blood/bile was reported in 40.5% and 16.7% of patients with HAV and HEV, respectively. The concordance rate between the anti-HEV-IgM results of both ELISA kits was 50%. HEV RNA was detected in only 17% of patients with HEV. The diagnosis of HEV needs clinical consideration due to incomplete HEV diagnostics.

## Introduction

Acute viral hepatitis (AVH) is an acute infection and/or replication of viruses in the liver that can induce liver injury of varying severity within a period of < 6 months. The etiology of AVH mainly includes infection of hepatitis A virus (HAV), hepatitis B virus (HBV), hepatitis C virus (HCV), and hepatitis E virus (HEV). Moreover, Epstein-Barr virus (EBV), cytomegalovirus (CMV), herpes simplex virus (HSV), can cause bystander hepatitis as a manifestation of systemic infection.

The estimated global incidence of AVH was 340 million in 2017^1^, and the morbidity and mortality caused by AVH pose a substantial threat to public health^1,2^. The burden of AVH is negatively associated with socioeconomic development status, with the highest burden in low-income countries. However, studies on AVH are scarce, especially in countries experiencing a rapid transition in socio-economic development.

The etiology, epidemiology, and clinical features of AVH have been undergoing dynamic alterations depending on the geographical region and vaccination strategy for viral hepatitis^3^. For example, HAV is mostly transmitted through the fecal–oral route by the consumption of contaminated food or water; however, it is common in sexually active homosexual men in developed countries, while the incidence of HBV infection continues to occur in countries with universal vaccination programs because of the increase in the number of immigrants from high-prevalence areas^4^. Moreover, awareness about HEV remains low, even among physicians, and there is no standard diagnostic method for HEV infection.

Thus, this prospective, nationwide multicenter study aimed to elucidate the etiology and clinico-epidemiological characteristics of AVH during 2020–2021 in South Korea. Additionally, the performance of different HEV diagnostic methods was comparatively analyzed using the hepatitis E patients’ blood samples.

## Materials and methods

### Participants

Acute hepatitis was defined as a condition with serum aspartate aminotransferase (AST) or alanine aminotransferase (ALT) levels > 200 IU/L in patients without evidence of aggravation of underlying chronic liver disease according to the investigators’ consensus for the purpose of this study. This nationwide study prospectively enrolled 428 patients aged > 18 years who were diagnosed with acute hepatitis at 12 university hospitals in South Korea between February 2020 and May 2021. Each patient provided informed written consent, and the study was approved by 12 institutional review boards of each institution.

**Figure 1:**
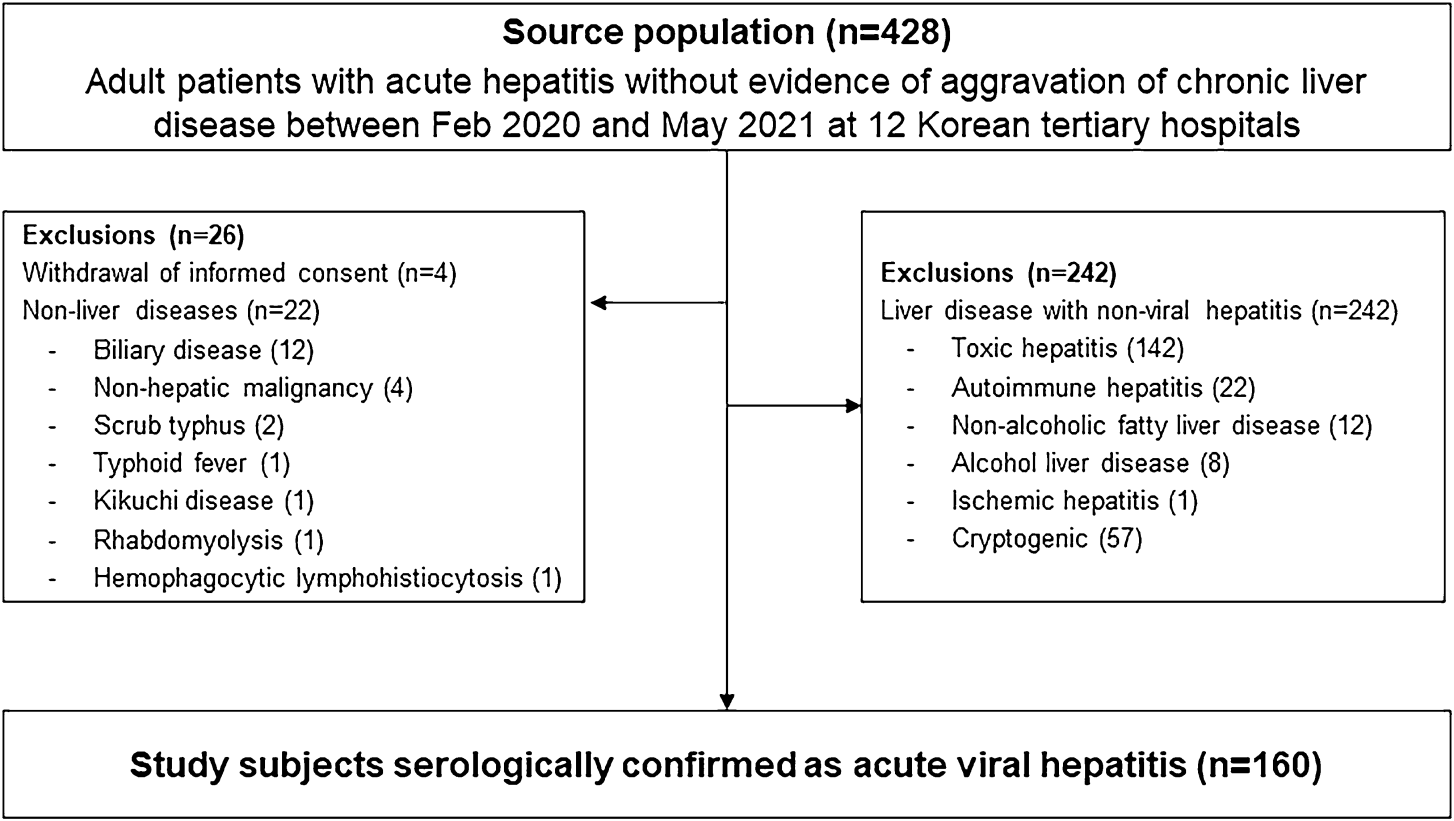
Flowchart illustrating the enrollment and exclusion of participants. This nationwide study prospectively enrolled 427 patients aged > 18 years who were diagnosed with acute hepatitis at 12 university hospitals in South Korea between February 2020 and May 2021. After excluding non-viral causes, 162 patients with serologically confirmed AVH were included in the study population.

After initial examination, 22 patients were excluded due to increased transaminase levels because of biliary disease, metastatic cancers, rhabdomyolysis, scrub typhus, typhoid fever, Kikuchi disease, and hemophagocytic lymphohistiocytosis. Moreover, 240 patients with acute hepatitis due to non-viral causes such as toxic hepatitis and autoimmune hepatitis, and 4 patients who withdrew consent for participation were excluded. Finally, 160 patients with serologically confirmed AVH were included (Fig. 1).

### Differential diagnosis of acute viral hepatitis

The etiology of AVH was defined as follows: hepatitis A by anti-HAV immunoglobulin M (IgM) positivity, hepatitis B by hepatitis B surface antigen (HBsAg) positivity and/or antibody for hepatitis B core antigen (anti-HBc) IgM positivity, hepatitis C by HCV RNA positivity with or without anti-HCV positivity, and hepatitis E by anti-HEV IgM positivity. Serological testing for hepatitis A, B, and C was performed using the methods that were approved and established at each hospital. The diagnosis of hepatitis E was based on a positive result for anti-HEV IgM by either of two enzyme-linked immunosorbent assays (ELISA) (Abia®, AB Diagnostic Systems, Berlin, Germany; Wantai BioPharm, Beijing, China), for qualitative determination of IgM-class antibodies to HEV. Considering the possibility of false positive or false negative results for anti-HEV IgM, the Abia anti-HEV IgM test was performed in the clinical setting (which is the only approved kit in South Korea), and the Wantai anti-HEV IgM test was additionally performed for research use. Furthermore, despite anti-HEV IgM positivity, cases with concurrently positive results for other viral hepatitis or autoimmune hepatitis were excluded from HEV infection, because of a high probability of false-positive results. Hepatitis virus D infection was not included in this study since it is very rare and no screening test is available in South Korea.

Acute hepatitis caused by other viruses was defined as follows: CMV hepatitis caused by anti-CMV IgM positivity, EBV hepatitis caused by anti-EBV viral capsid antigen (VCA)-IgM positivity with reactive lymphocytosis, and HSV hepatitis caused by anti-HSV IgM positivity.

### Comparison of diagnostic methods for HEV using blood obtained from patients with hepatitis E

Anti-HEV IgM and IgG testing using the Abia kit were performed for diagnosis of HEV hepatitis in the clinical setting. For whom with non-A, non-B, and non-C AVH patients, the blood samples were collected in a serum separation tube, centrifuged within 2 h, refrigerated, and safely transported to a central laboratory (Seoul Central Laboratory) within 24 h. Considering the possibility of false positive or false negative for ELISA, HEV IgM and IgG was tested again using the Wantai ELISA kit. In case of either Abia or Wantia HEV IgM positivity, a real-time quantitative polymerase chain reaction (qPCR) test were performed to detect HEV RNA. Briefly, viral RNA was extracted from the serum samples of patients with HEV using the QIAamp® viral RNA mini kit (Qiagen, Germany), and reverse transcription (RT)-qPCR amplification was performed using the TaqMan assay with a CFX96 Dx system (Bio-Rad Laboratories, CA, USA). The primers and probes used in this study and detailed methodology of the RT-qPCR test are shown in Supplementary Table 1.

### Data collection at baseline and follow-up

Upon enrollment in this study, trained research coordinators at each of the 12 hospitals interviewed the patients using a standardized questionnaire, which included demographic and socioeconomic status (age, sex, body mass index [BMI], education level, and occupation), health behaviors (smoking, alcohol intake, medications, and use of herbal or various other health supplements), comorbidities, and exposure to risk factors for hepatitis A, B, C, or E within 3 months of AVH diagnosis (history of traveling abroad, known high-risk foods for HAV or HEV such as undercooked seafood or raw meat, history of sexual intercourse with people with unknown hepatitis status, history of invasive procedures, and so on).

Laboratory data were collected from the electronic medical records of patients, which included the complete blood count, blood urea nitrogen, serum creatinine, prothrombin time, total protein, albumin, total bilirubin, alkaline phosphatase, gamma-glutamyl transferase, AST, and ALT levels. Information on clinical outcomes, such as hospitalization, intensive care unit treatment, presence of acute hepatic failure, liver transplantation, and survival, was collected during a median follow-up period of 6 months.

Research coordinators entered all data into the established electronic case report form (eCRF) on the web page (http://www.acutehep.or.kr). The coordinators underwent repeated education based on the original and updated data guidelines, and data cleaning, in addition to a bimonthly correction of detected errors and subsequent monitoring, was performed by an independent professional data manager to manage the quality of data provided by multiple centers.

### Statistical analysis

A descriptive analysis was performed for demographic and clinic-epidemiological characteristics. The etiological distribution of AVH was represented as numbers and frequency (%). Categorical variables were compared using the chi-squared or Fisher’s exact test. Numerical variables with normal and non-normal distribution were compared using Student’s *t* test and Mann–Whitney *U* test, respectively. A one-way analysis of variance was used to compare ≥ 3 groups. A p-value < 0.05 was considered statistically significant. All analyses were conducted using SPSS for Windows version 26.0 (SPSS Inc., Chicago, IL, USA).

### Ethical approval

Each patient provided informed written consent, and the study was approved by 12 institutional review boards of each institution (Seoul National University Bundang Hospital IRB, Chungnam National University Hospital IRB, Soonchunhyang University Bucheon Hospital IRB, Inje University Busan Paik Hospital IRB, Inje University Ilsan Paik Hospital IRB, Jeonbuk National University Hospital IRB, Chonnam National University Hwasun Hospital IRB, Chonnam National University Hospital IRB, Kangwon National University Hospital IRB, Keimyung University Dongsan Hospital IRB, Gyeongsang National University Hospital IRB, and Jeju National University Hospital IRB). This study was conducted in accordance with the Helsinki Declaration in 1975 (revised in 2000). The protocol of this study was approved by the Institutional Review Board of each hospital.

## Results

### Etiologic proportion, comparison of clinical features, and outcomes of AVH according to etiology

**Figure 2:**
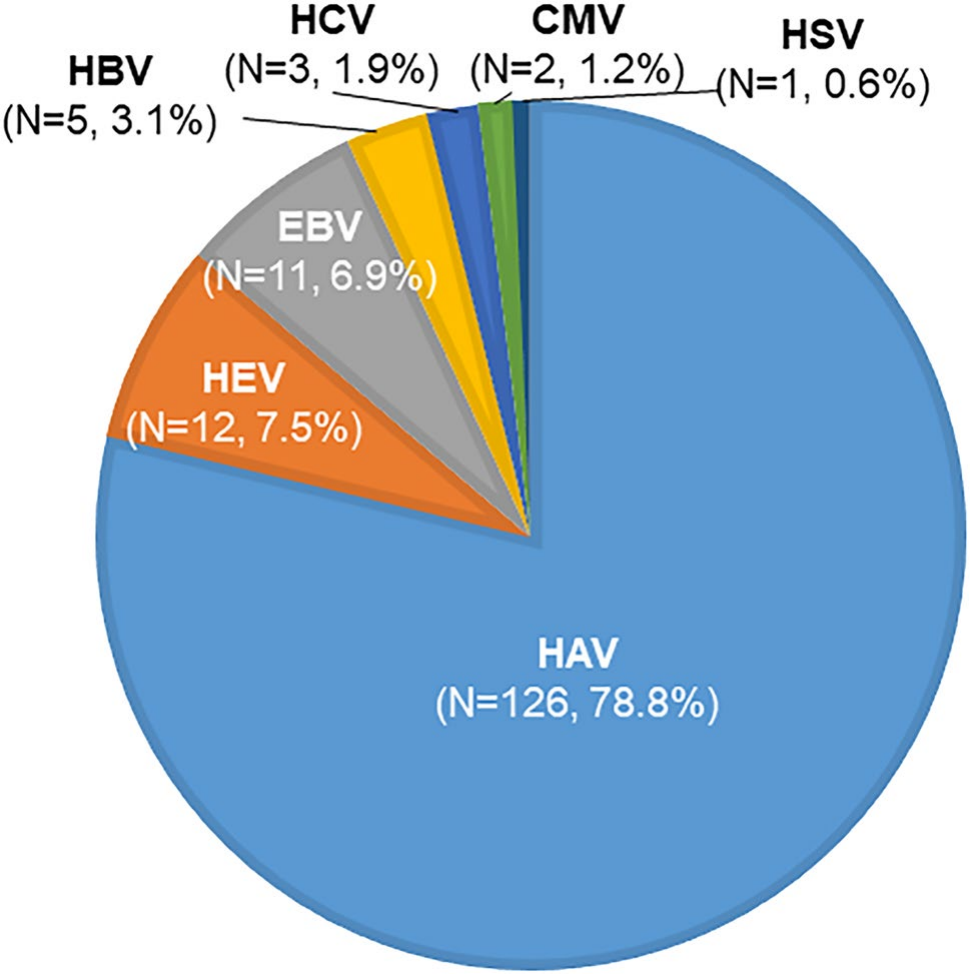
Proportion of the etiology of acute viral hepatitis in South Korea (2020–2021). Among 192 patients with AVH, HAV was the most common cause (77.2%) of AVH, followed by HEV (10.5%), HBV (3.1%), and HCV (1.9%). EBV, CMV, and HSV accounted for 5.6%, 1.2% and 0.6% of AVH cases due to non-hepatotropic viruses, respectively. *HAV* hepatitis A virus, *HBV* hepatitis B virus, *HCV* hepatitis C virus, *HEV* hepatitis E virus, *EBV* Epstein-Barr virus, *CMV* cytomegalovirus and *HSV* Herpes-simplex virus.

The study population included 160 patients with AVH (median age, 43 years). The proportion of male patients was 52.5%, and 95.0% of patients had symptomatic manifestation. HAV was the most common cause (78.8%) of AVH, followed by HEV (7.5%), HBV (3.1%), and HCV (1.9%). Among the other viruses, EBV, CMV, and HSV accounted for 6.9%, 1.2%, and 0.6% of AVH cases, respectively. The etiologic distribution of AVH is depicted in Fig. 2.

The patients’ clinical characteristics based on the etiology of AVH are shown in Table 1. Patients with HAV presented with a lower median age (43 years), greater symptomatic manifestation, higher levels of bilirubin, AST, and ALT, and a lower frequency of comorbidities than patients with other etiologies. The median age of patients with HEV was 57 years with a male predominance, and relatively low levels of bilirubin, AST, and ALT suggesting mild clinical hepatitis. The median age of patients with HBV and HCV was 53 and 65 years, respectively; the levels of bilirubin, AST, and ALT were higher in the HBV group than in the HCV group. Interestingly, all 3 patients with acute hepatitis C were women, and 2 patients were asymptomatic.

**Table 1:** Comparison of clinical characteristics according to cause of acute viral hepatitis.

	HAV (n = 126)	HBV (n = 5)	HCV (n = 3)	HEV (n = 12)	Non-hepatotropic virus (n = 14)	*P*-value
Age (years)	43 (38–48)	53 (49–62)	65 (57–69)	57 (48–66)	27 (25–32)	< 0.001
Male sex	71 (56.3%)	2 (40%)	0 (0%)	8 (66.7%)	3 (21.4%)	0.028
Body mass index (kg/m^2^)	23.9 (21.3–26.5)	22.8 (21.3–23.1)	24.2 (22.9–24.3)	23.0 (21.3–27.8)	21.5 (19.7–23.5)	0.176
Underlying diseases^†^	16 (12.8%)	1 (20%)	2 (66.67%)	5 (41.7%)	0 (0%)	< 0.001
Diabetes	11 (8.7%)	1 (20%)	2 (66.67%)	4 (33.3%)	0 (0%)	0.001
Chronic kidney disease	1 (0.8%)	0 (0%)	1 (33.3%)	0 (0%)	0 (0%)	< 0.001
Myocardial infarction	2 (1.6%)	0 (0%)	0 (0%)	0 (0%)	0 (0%)	0.970
Heart failure	1 (0.8%)	0 (0%)	0 (0%)	0 (0%)	0 (0%)	0.992
Stroke	1 (0.8%)	0 (0%)	0 (0%)	1 (8.3%)	0 (0%)	0.256
Respiratory disease	1 (0.8%)	0 (0%)	0 (0%)	0 (0%)	0 (0%)	> 0.999
Symptoms						
Symptomatic	124 (98.4%)	3 (60%)	1 (33.3%)	10 (83.3%)	14 (100%)	< 0.001
Fever	77 (61.1%)	1 (20%)	0 (0%)	2 (16.7%)	9 (64.3%)	0.003
Chills	83 (65.9%)	1 (20%)	0 (0%)	6 (50%)	8 (57.1%)	0.036
Anorexia	87 (69%)	2 (40%)	0 (0%)	6 (50%)	11 (78.6%)	0.035
Nausea	92 (73%)	3 (60%)	0 (0%)	6 (50%)	8 (57.1%)	0.031
Myalgia	81 (64.8%)	3 (60%)	0 (0%)	5 (41.7%)	8 (66.6%)	0.008
Jaundice	84 (66.7%)	3 (60%)	1 (33.3%)	8 (66.7%)	3 (21.4%)	0.016
Laboratory findings						
WBC (/mm^3^)	4980 (3660–6590)	5340 (5260–5500)	4010 (3800–4975)	5900 (3500–8500)	8350 (5610–14,210)	< 0.001
Hemoglobin (g/dL)	14.8 (13.8–15.9)	14.8 (12.7–14.9)	12.6 (12.1–13.1)	13.7 (12.5–17.1)	13.6 (13.1–4.5)	0.189
Platelet (× 1000/mm^3^)	171 (135–212)	185 (167–200)	192 (165–201)	126 (98–208)	200 (190–256)	0.364
BUN (mg/dL)	11 (8–14)	13 (11–13)	12 (10–16)	15 (10–26)	10.5 (8–12.5)	0.425
Creatinine (mg/dL)	0.74 (0.62–0.90)	0.64 (0.63–0.79)	0.61 (0.6–2.38)	0.83 (0.60–1.24)	0.63 (0.52–0.76)	0.645
Prothrombin time (INR)	1.13 (1.03–1.29)	1.09 (1.02–1.15)	0.98 (0.98–1.02)	1.00 (0.94–1.12)	1.01 (0.95–1.14)	0.557
Total bilirubin (mg/dL)	4.33 (1.96–6.29)	2.68 (2.37–4.62)	1.10 (1.05–1.14)	2.90 (0.64–11.61)	0.86 (0.66–1.62)	0.004
Peak level^‡^	5.34 (3.16–7.25)	4.62 (2.37–4.64)	1.17 (1.08–2.41)	2.90 (0.64–12.64)	0.95 (0.76–1.62)	0.003
AST (IU/L)	1500 (587–1500)	1395 (1266–1500)	519 (383–698)	475 (288–892)	518 (396–750)	< 0.001
Peak level^‡^	1500 (695–1500)	1500 (1266–1500)	877 (562–1023)	541 (319–1197)	584 (396–750)	< 0.001
ALT, initial (IU/L)	2250 (1467–2250)	2250 (1731–2250)	922 (510–1037)	861 (600–1372)	540 (434–899)	< 0.001
Peak level^‡^	2250 (1591–2250)	2250 (1731–2250)	1150 (626–1395)	933 (606–1423)	615 (435–995)	< 0.001
ALP (IU/L)	213 (165–268)	223 (172–261)	111 (100–171)	189 (135–315)	155 (88–322)	0.641
GGT (IU/L)	384 (262–571)	350 (300–556)	203 (160–246)	375 (58–601)	141 (105–256)	0.013
Clinical outcomes						
Hospitalization	112 (90.3%)	2 (40%)	2 (66.7%)	11 (91.7%)	10 (71.4%)	0.004
Liver biopsy	0 (0%)	0 (0%)	1 (33.3%)	1 (8.3%)	2 (14.3%)	< 0.001
Critical care	1 (0.8%)	0 (0%)	0 (0%)	0 (0%)	0 (0%)	0.992
Hemodialysis	4 (3.2%)	0 (0%)	0 (0%)	1 (8.3%)	0 (0%)	0.785
Encephalopathy	1 (0.8%)	0 (0%)	0 (0%)	1 (8.3%)	0 (0%)	0.263
Liver transplant	0 (0%)	0 (0%)	0 (0%)	0 (0%)	0 (0%)	> 0.999
Mortality within 90 days	0 (0%)	0 (0%)	0 (0%)	0 (0%)	0 (0%)	> 0.999

Values are presented as median (range) or n (%). The upper detection limits of AST and ALT levels are 1500 and 2250 IU/L, respectively. The test results of laboratory findings are all initial test results, except for the peak levels of total bilirubin, AST, and ALT.

*WBC* white blood cell, *BUN* blood urea nitrogen, *INR* international normalized ratio, *ALP* alkaline phosphatase, *GGT* gamma-glutamyl transferase, *AST* aspartate transaminase and *ALT* alanine transaminase.

^†^Patients with one or more underlying diseases

^‡^The peak levels of total bilirubin, AST, and ALT are the highest following enrollment.

The clinical characteristics of patients with other viral hepatitis are depicted in Table 2. The median age of patients with EBV was lower (26 years) than that of patients with CMV (30–43 years) or HSV (61 years), with a high female predominance. They all complained of symptoms such as fever and anorexia, and required hospital admission in 75%, but none received intensive care or liver transplantation. Patients with EBV showed higher AST and ALT levels than patients with CMV. No patient was immunocompromised.

**Table 2:** Comparison of the clinical characteristics of acute viral hepatitis caused by non-hepatotropic viruses.

	Epstein-barr virus (n = 11)	Cytomegalovirus (n = 2)	Herpes simplex virus (n = 1)
Age (years)	26 (24–28)	30–43	61
Male sex	3 (27.3%)	0 (0%)	0 (0%)
Body mass index (kg/m^2^)	22.8 (19.7–23.8)	19.2–21.3	21.6
Underlying diseases*	0 (0%)	0 (0%)	0 (0%)
Immunosuppressed status^†^	0 (0%)	0 (0%)	0 (0%)
Symptoms			
Symptomatic	11 (100%)	2 (100%)	1 (100%)
Fever	8 (72.7%)	1 (50%)	0 (0%)
Chills	7 (63.6%)	1 (50%)	0 (0%)
Anorexia	10 (90.9%)	0 (0%)	1 (100%)
Nausea	7 (63.6%)	1 (50%)	0 (0%)
Myalgia	9 (81.8%)	1 (50%)	0 (0%)
Jaundice	3 (27.3%)	0 (0%)	1 (100%)
Laboratory findings, initial			
WBC (/mm^3^)	7010 (5320–12,420)	7010–13,750	5320
Neutrophil, %	35 (16.8–44.2)	42.3–92.7	44.2
Lymphocyte, %	54.5 (31–76.4)	3.7–49.8	35.9
NLR	0.668 (0.22–1.23)	0.85–25.05	1.23
Hemoglobin (g/dL)	13.6 (13.1–14.6)	11.8–13.7	13.0
Platelet (× 1000/mm^3^)	199 (190–259)	118–297	259
BUN (mg/dL)	11 (8–12)	7–14	14
Creatinine (mg/dL)	0.69 (0.59–0.80)	0.62–0.64	0.59
Prothrombin time (INR)	1.01 (0.95–1.15)	0.96–1.11	0.99
Total bilirubin (mg/dL)	0.95 (0.73–2.67)	0.52–1.15	0.70
Peak level^‡^	0.95 (0.76–2.67)	0.69–1.15	0.94
AST (IU/L)	457 (316–949)	316–330	397
Peak level^‡^	579 (391–949)	316–391	397
ALT, initial (IU/L)	547 (480–874)	200–483	974
Peak level^‡^	674 (483–1056)	385–483	974
ALP (IU/L)	241 (137–385)	168–385	81
GGT (IU/L)	154 (107–283)	126–229	136
Serologic test, positive rate			
EBV VCA IgM	11 (100%)	0 (0%)	0 (0%)
CMV IgM	0 (0%)	2 (100%)	0 (0%)
HSV IgM	0 (0%)	0 (0%)	1 (100%)
HEV IgM	2 (18.2%)	0 (0%)	0 (0%)
Clinical outcomes			
Hospitalization	7 (63.6%)	1 (50%)	1 (100%)
Liver biopsy^§^	0 (0%)	1 (50%)	1 (100%)
Critical care or dialysis	0 (0%)	0 (0%)	0 (0%)
Mortality or transplantation within 90 days	0 (0%)	0 (0%)	0 (0%)

Values are presented as median (range) or n (%).

The upper detection limits of AST and ALT levels are 1500 and 2250 IU/L, respectively. The test results of laboratory findings are all initial test results, except for the peak levels of total bilirubin, AST, and ALT.

*WBC* white blood cell, *NLR* neutrophil–lymphocyte ratio, *BUN* blood urea nitrogen, *INR* international normalized ratio, *ALP* alkaline phosphatase, *GGT* gamma-glutamyl transferase, *AST* aspartate transaminase, *ALT* alanine transaminase, *EBV* Epstein-Barr virus, *CMV* Cytomegalovirus, *HSV* Herpes simplex virus, *VCA* virus capsid antigen, *PCR* polymerase chain reaction, *IHC* immunohistochemistry and *NT* no test.

*Underlying diseases, including myocardial infarction, heart failure, diabetes, chronic kidney disease, chronic respiratory disease, stroke, or rheumatologic disease.

^†^Immunosuppressed status, including cancer patients receiving chemotherapy, solid organ transplant recipients, HIV patients, or patients taking immunosuppressive drugs.

^‡^The peak level of total bilirubin, AST, and ALT are the highest following enrollment.

^§^Predominant lymphocytic infiltration was observed in the portal tract and sinusoid in both patients who underwent liver biopsy, but plasma cells were not observed. Immunohistochemical staining for each virus was negative.

Of the 160 patients with AVH, 137 (86.7%) needed hospitalization, 5 (3.2%) needed hemodialysis, and 1 (0.6%) patient required intensive care unit treatment. Two patients (1.3%) developed acute liver failure; specifically, one 53-year-old man with HAV and one 63-year-old man with HEV, neither of whom had a history of underlying chronic liver disease. There was no instance of mortality, and almost all patients recovered within 3 months of diagnosis. There was no case of progression to chronic hepatitis among the HBV, HCV, and HEV groups at least 6 months follow-up. In 5 cases with acute hepatitis B and 3 cases with acute hepatitis C, all of them recovered rapidly without DAA therapy.

### Risk factors of patients with AVH according to etiology

A selective summary of the questionnaire survey on the risk factors related to HAV and HEV showed that patients with HEV tended to be engaged in livestock-related occupations, such as butchers, more frequently, and provided significantly higher positive responses to questions enquiring about the intake of the blood or bile of wild boars (16.7 vs. 0%, p = 0.007) within 3 months before diagnosis compared to the HAV group. In contrast, patients with HAV provided significantly higher positive responses to questions enquiring about the intake of uncooked clams or oysters (40.5% versus 8.3%, p = 0.031) compared to patients with HEV (Table 3).

**Table 3:** Selected summary of a questionnaire survey on the risk factors related to HAV compared to HEV and HBV compared to HCV.

	HAV (n = 126)	HEV (n = 12)	*P*-value
Occupation			
Livestock-related	2 (1.6%)	2 (16.7%)	0.007
Food intake (within 3 months)			
Uncooked clams or oyster	51 (40.5%)	1 (8.3%)	0.031
Sashimi	76 (60.3%)	4 (33.3%)	0.123
Sushi	64 (50.8%)	5 (41.7%)	0.764
Salted fish	64 (50.8%)	5 (41.7%)	0.764
Liver and intestines of cattle	3 (2.4%)	1 (8.3%)	0.308
Uncooked pork	10 (7.9%)	0 (0%)	0.600
Uncooked beef	28 (22.2%)	3 (25%)	0.732
Blood or bile of wild animals^†^	0 (0%)	2 (16.7%)	0.007
Jamón or salami	4 (3.2%)	0 (0%)	> 0.999
Dried fruit^‡^	9 (7.1%)	3 (25.0%)	0.071

	HBV^§^ (n = 5)	HCV (n = 3)	
Risk behaviors			
Procedure or surgery	2 (40%)	0	
Tattoo or piercing	1 (20%)	0	
Acupuncture	1 (20%)	0	
Blood transfusion	0	0	
Syringe stab	0	0	
Sex with an unknown person	0	0	

Values are presented as n (%).

*HAV* hepatitis A virus, *HBV* hepatitis B virus, *HCV* hepatitis C virus and *HEV* hepatitis E virus.

^†^Two patients with HEV ingested wild boar blood or bile.

^‡^The types of dried fruits included pineapple, mango, apple, strawberry, blueberry and cranberry, and the intake history of products sold in the market after processing and packaging.

^§^Among patients with HBV, one patient underwent orthopedic surguery (internal fixation), another patient underwent a dental implant procedure and acupuncture, and the other patient underwent tattooing.

The selective summary of the questionnaire survey on the risk factors related to HBV and HCV showed that 3 of the 5 patients with HBV underwent invasive procedures, such as dental implant placement and acupuncture (n = 1), orthopedic surgery (n = 1), and tattooing (n = 1) within 3 months of AVH diagnosis. However, no suspected risk factor was observed among 3 patients with acute hepatitis C (Table 3).

### Comparison of the diagnostic tests for acute hepatitis E

The Laboratory results of the 12 patients with HEV and 5 patients with false-positive HEV who underwent comparison of the two types of ELISA tests for anti-HEV IgM and real-time RT PCR for HEV RNA are summarized in Table 4. Ten of the 12 patients who were finally diagnosed with acute hepatitis E were tested for anti-HEV IgM with two diagnostic kits, Abia and Wantai, because one patient did not consent to blood sampling, and the other patient wished to undergo only the Wantai test since he did not have to pay for it. Five of these 10 patients (50%) showed concordant positivity with both ELISA kits, and 2 of these 5 patients showed HEV RNA positivity.

**Table 4:** Clinico-epidemiologic characteristics and comparative results of the diagnostic tests of patients positive for anti-HEV IgM.

No	Sex	Age	Anti-HEV IgM		Anti-HEV IgG		HEV RNA	TB (mg/dL)	AST (IU/L)	ALT (IU/L)	Occupation	Final diagnosis
			Abia	Wantai	Abia	Wantai	RT-PCR					
1	M	53	( +)	( +)	( +)	( +)	(–)	6.96	1500	2250	Official	HEV
2	M	74	( +)	( +)	( +)	( +)	(–)	13.15	263	933	Inoccupation	HEV
3	M	70	( +)	( +)	( +)	( +)	( +)	6.98	945	1133	Inoccupation	HEV
4	M	58	( +)	( +)	NT	( +)	( +)	2.0	1281	2250	Office administration	HEV
5	M	60	( +)	( +)	NT	( +)	(–)	23.49	673	1423	Inoccupation	HEV
6	M	63	( +)	(–)	( +)	( +)	(–)	46.94	576	767	Livestock raising	HEV
7	F	53	( +)	(–)	( +)	( +)	(–)	0.8	285	600	Teacher	HEV
8	F	40	( +)	(–)	( +)	( +)	(–)	0.58	112	217	Housewife	HEV
9	F	46	( +)	(–)	( +)	( +)	(–)	0.43	1500	1218	Agriculture	HEV
10	F	39	( +)	(–)	( +)	( +)	(–)	0.17	505	622	Office administration	HEV
11	M	47	( +)	NT	(–)	NT	NT	1.34	444	435	Manufacturing	HEV
12	M	65	NT	( +)	NT	( +)	(–)	3.8	420	789	Livestock raising	HEV
13*	M	52	( +)	(–)	NT	(–)	(–)	1.01	161	301	Office administration	AIH^†^
14*	M	77	(–)	( +)	NT	(–)	(–)	7.97	995	743	Inoccupation	AIH^†^
15*	F	80	( +)	(–)	(–)	(–)	(–)	1.92	220	38	Inoccupation	AIH^†^
16*	F	24	( +)	(–)	NT	(–)	(–)	0.95	203	386	Manufacturing	EBV
17*	F	26	( +)	(–)	(–)	(–)	(–)	0.5	683	532	Inoccupation	EBV

*No*. number of patients, *RT*-*PCR* reverse transcription polymerase chain reaction, *TB* total bilirubin, *AST* aspartate transaminase, *ALT* alanine transaminase, *HEV* hepatitis E virus, *HAV* hepatitis A virus, *AIH* autoimmune hepatitis, *EBV* Epstein-Barr virus and *NT* not tested.

The upper detection limits of AST and ALT were 1500 and 2250 IU/L, respectively.

*Number 13–17 indicated cases showing the false-positive results for anti-HEV IgM.

^†^Diagnosis of AIH was based on liver biopsy results and other diagnostic criteria. According to the simplified criteria for AIH, number 13 and 14 were probable AIH, number 15 was definite AIH.

## Discussion

This nationwide prospective study found that HAV was the most common cause (78.8%) of AVH during 2020–2021 in South Korea, followed by HEV (7.5%), while HBV and HCV accounted for 5% of HAV cases, and other viruses, such as EBV, CMV, and HSV, accounted for 8.7% of AVH cases according to the final diagnosis. Patients with HAV exhibited median age of 43 years, higher levels of ALT and bilirubin, higher hospitalization rates, and a higher frequency of undercooked shellfish or oyster intake compared to patients with other etiologies of AVH. Patients with HEV was characterized by median age of 50 years, milder hepatitis and a higher proportion of intake history of bile or blood of wild animals than HAV patients. Patients with HEV accounted 7.5% of AVH, showing a median age of 26 years and characteristic clinical features of infectious mononucleosis. Among 160 AVH cased who visited to tertiary hospitals, the incidence of acute liver failure was 1.3%, but there was no instance of mortality. HEV diagnostic tests require further improvement, with emphasis on the careful interpretation of serological tests for HEV.

HAV was included in the Expanded Program on Immunization (EPI) since 2015 in South Korea^5^. The seroprevalence of HAV in a large tested population was 88.7% in 2019 in individuals aged 10–19 years, 32.8% in individuals aged 20–29 years, 32.4% in individuals aged 30–39 years, 63.2% in individuals aged 40–49 years, and > 94.2% in individuals aged > 50 years. Therefore, the current adult population aged 20–49 years is the most susceptible to HAV infection. In this study, the median age of patients with HAV was 43 years, most of whom were severely symptomatic and required hospitalization. The history of ingestion of clams and oysters was higher in patients with HAV than in patients with HEV in this study, suggesting that contaminated shellfish still constitute a high-risk food item for HAV in Korea.

HBV vaccination was included in EPI since 1995, and the age-standardized HBsAg prevalence among individuals aged > 18 years was 2.8% in 2018 in South Korea^6^. In this study, the median age of patients with HBV was 53 years, suggesting a vaccine-missed age group, and the etiological proportion of HBV was only 3%.

The prevalence of anti-HCV among the adult Korean population decreased from 0.78% in 2009 to 0.6% in 2015, whereas anti-HCV prevalence increased in an age-dependent manner to over 1% among the population aged > 50 years^7^. Similarly, this study showed that the median age of patients with acute hepatitis C was 65 years, and HCV accounted for only 2% of AVH cases.

HEV infection is highly prevalent in individuals aged > 40 years in the Korean population, although more than 90% of HEV infections are asymptomatic. Anti-HEV IgG prevalence in the Korean population was reportedly 0.2%, 1.2%, 2.4% 12.0%, and 20.9% in individuals aged 10–19, 20–29, 30–39, 40–49, and 50–55 years in 2007–2009, respectively^8,9^. In this study, HEV is more common etiology of AVH than HBV and HCV. The median age of patients with HEV was 57 years, and clinical manifestation was milder than that of HAV, albeit with a relatively higher frequency of acute liver failure.

There is no gold standard test for the diagnosis of acute hepatitis E^10^. Ideally, the definitive diagnosis of HEV is based on the detection of HEV RNA in serum or stool; however, the level of viremia plummeted abruptly after symptom development in patients with HEV, as shown in this study, since the serum tested positive for HEV RNA in only 17% of patients. Anti-HEV IgM positivity was considered to be a diagnostic criterion for HEV infection, especially in immune-competent patients. The two ELISA kits analyzed in this study showed a concordance rate of merely 50%, which is far from standardization. Because various antigens are used, depending on the manufacturer of the ELISA test, the agreement rate of results is 32.6–71%^11–14^, as clearly demonstrated in this study. Therefore, seroprevalence studies on HEV infection should be carefully interpreted according to used ELISA kit and subjects’ demographics. Moreover, the problem of false positivity of anti-HEV IgM was previously reported in patients with EBV or CMV infection^15^ and HAV infection^16^, which probably arises due to cross-reactivity. In our study, there were 3 cases of AIH and 2 cases with EBV hepatitis showing anti-HEV IgM positivity with either the Abia or Wantai ELISA kit. However, case 11 and case 12 in the Table 4 showed a positive anti-HEV IgM in only one kit without any other cause of hepatitis, so that they were properly classified as HEV hepatitis, according to predefined diagnostic criteria. HEV is implicated as a trigger for the development of AIH, and several case reports have described overlapping between the clinical features of AIH and acute hepatitis E^17^. Therefore, the lack of standardization of diagnostic methods of HEV should be urgently remedied.

This study showed that symptomatic hepatitis due to EBV with or without infectious mononucleosis is as common as HEV hepatitis, though it is known to be rare. Although EBV does not infect hepatocytes, cytotoxic T cells recognizing EBV infected B-cells cause collateral damage to the hepatocytes^18^. EBV hepatitis may present as cholestatic hepatitis with a rare case of vanishing bile duct syndrome or autoimmune and granulomatous hepatitis. The seropositivity rate of EBV decreased from 89.4% during 2000–2008 to 76.2% during 2009–2017 in the Korean population, and the overall seropositivity in individuals aged 10–19 years was 75.8% during 2000–2017^19,20^. The age of onset of EBV infection increases according to socio-economic development. In this study, the median age of EBV infection onset with symptomatic hepatitis was 26 years.

CMV hepatitis is very rare, especially in immunocompetent hosts, and can manifest as infectious mononucleosis^21,22^. Similarly, herpes simplex hepatitis is very rare, especially in immunocompetent hosts. In this study, one patient with CMV hepatitis and another with HSV hepatitis underwent liver biopsy, but the results of CMV and HSV immunostaining were negative for both.

This study has several limitations. First, the study population included patients who visited tertiary hospitals, resulting in referral bias as shown as the hospitalization rate of 86%. Second, detailed information related to sexual-habit-related risk factors was not included in the questionnaires, because sexual transmission of HEV has been described in men having sex with men^10^. Third, our in-house HEV RNA qPCR was not standardized using WHO standard of HEV samples. Given that HEV RNA in blood appears an early phase of acute hepatitis E, we speculate that our cases were mostly symptomatic and sometime delay from sample collection to PCR testing may explain the low positivity of HEV RNA in qPCR. Forth, HEV RNA was not tested in stool samples, which is reported to possess higher sensitivity than serum. However, the strength of this study lies in its multicenter, prospective design, as well as its well-prepared eCRF and comprehensive survey on the epidemiology of AVH using standardized questionnaires, reportage of follow-up of outcomes, and high-quality data. Additionally, we tested anti-HEV using two different anti-HEV kits and HEV RNA testing to investigate the diagnostic concordance for HEV.

In conclusion, HAV is currently the most common etiology of AVH, followed by HEV and EBV. Acute liver failure developed in 1% of AVH cases. The standardization of HEV diagnostics and emphasis on meticulous serological interpretation are imperative.

## Supplementary Information

Supplementary Information.

## Data Availability

The datasets used and/or analysed during the current study available from the corresponding author on reasonable request.

## Abbreviations

ALP: Alkaline phosphatase
ALT: Alanine aminotransferase
anti-HBc: Antibody for hepatitis B core antigen
AST: Aspartate aminotransferase
AVH: Acute viral hepatitis
BMI: Body mass index
BUN: Blood urea nitrogen
CMV: Cytomegalovirus
EBV: Epstein-Barr virus
eCRF: Electronic case report form
ELISA: Enzyme-linked immunosorbent assay
EPI: Expanded program on immunization
GGT: Gamma-glutamyl transferase
HAV: Hepatitis A virus
HBsAg: Hepatitis B surface antigen
HBV: Hepatitis B virus
HCV: Hepatitis C virus
HEV: Hepatitis E virus
HSV: Herpes simplex virus
IgM: Immunoglobulin M
IHC: Immunohistochemistry
INR: International normalized ratio
NLR: Neutrophil–lymphocyte ratio
RT-qPCR: Reverse transcription-quantitative polymerase chain reaction
VCA: Viral capsid antigen
WBC: White blood cell
