# Author Correction: Etiology and clinical characteristics of acute viral hepatitis in South Korea during 2020–2021: a prospective multicenter study

**DOI:** 10.1038/s41598-024-78816-2

**Published:** 2024-11-19

**Authors:** Chan Young Jeong, Gwang Hyeon Choi, Eun Sun Jang, Young Seok Kim, Youn-Jae Lee, In Hee Kim, Sung Bum Cho, Jae Hyun Yoon, Kyung-Ah Kim, Dae Hee Choi, Woo Jin Chung, Hyun Chin Cho, Seong Kyun Na, Yun-Tae Kim, Byung Seok Lee, Sook-Hyang Jeong

**Affiliations:** 1 Departments of Internal Medicine, Seoul National University Bundang Hospital, Seoul National University College of Medicine, 82 Gumi-ro 173 Beon-gil, Bundang-gu, Seongnam, 13620 Republic of Korea; 2 Department of Internal Medicine, Soonchunhyang University Bucheon Hospital, Bucheon, Republic of Korea; 3 Department of Internal Medicine, Inje University Busan Paik Hospital, Busan, Korea; 4 Department of Internal Medicine, Jeonbuk National University Hospital, Jeonju, Republic of Korea; 5 Department of Internal Medicine, Chonnam National University Hwasun Hospital, Hwasun, Republic of Korea; 6 Department of Internal Medicine, Chonnam National University Hospital, Gwangju, Republic of Korea; 7 Departments of Internal Medicine, Inje University Ilsan Paik Hospital, Goyang, Republic of Korea; 8 Departments of Internal Medicine, Kangwon National University Hospital, Chunchon, Republic of Korea; 9 Departments of Internal Medicine, Keimyung University Dongsan Hospital, Daegu, Republic of Korea; 10 Departments of Internal Medicine, Gyeongsang National University Hospital, Jinju, Republic of Korea; 11 Departments of Internal Medicine, Jeju National University Hospital, Jeju, Republic of Korea; 12 Seoul Clinical Laboratories, Yongin, Republic of Korea; 13 Departments of Internal Medicine, Chungnam National University Hospital, 282 Munhwa-ro, Jung-gu, Daejeon, 35015 Republic of Korea

Correction to: *Scientific Reports* 10.1038/s41598-023-40775-5, published online 31 August 2023

The original version of this Article contained an error in the spelling of the author Hyun Chin Cho which was incorrectly given as Hyun-Jin Cho.

The original Article has been corrected.

