# Supplementary material for: Etiology and clinical characteristics of acute viral hepatitis in South Korea during 2020–2021: a prospective multicenter study
Source: Sci Rep. 2023 Aug 31;13:14271. doi: 10.1038/s41598-023-40775-5 (PMC10471758; doi:10.1038/s41598-023-40775-5)
Supplement: Supplementary file 1 — Supplementary Information. [file 41598_2023_40775_MOESM1_ESM.docx]

**Supplementary Table 1. Nucleotide sequence of primers and probes for the detection of hepatitis E virus RNA and internal control plasmid using a reverse transcription (RT)-quantitative polymerase chain reaction (qPCR) test**

| **Target** | **Type** | **Sequence (5`→3`)** | **Product size (bp)** |
| --- | --- | --- | --- |
| HEV ORF 2/3 | Forward | CCGGCGGTGGTTTCTGG | 72 |
|  | Reverse | GGTTGGTTGGATGAATATAGG |  |
|  | Probe | FAM-CGAAGGGCTGAGAATCAACCC-BHQ1 |  |
|  |  |  |  |
| IC | Forward | GACATCGATATGGGTGCCG | 141 |
|  | Reverse | CGAGACGATGCAGCCATTC |  |
|  | Probe | HEX-CTCATGCGTCTCCCT-BHQ1 |  |

HEV, hepatitis E virus; ORF, open reading frame; IC, internal control

The initial cDNA synthesis step was performed at 50°C for 10 min, followed by denaturation at 95°C for 10 min. DNA was amplified with 40 10-s cycles at 95°C and 40 30-s cycles at 59°C. Primers and probes were designed manually based on the multiple sequence alignment of HEV genome sequences in the ORF2/3 region commonly used in a real-time PCR test. The following sequences were aligned using the Clustal Omega program to identify highly conserved regions. The specificity of each primer and probe was determined via a BLAST search against the NCBI database. An internal control plasmid was constructed with synthetic DNA at Macrogen Inc., Seoul, Korea. The sequence of the internal control plasmid bore no significant nucleotide similarity to any known, naturally occurring, PCR-amplifiable nucleotide sequences reported in the NCBI database.
